# Effectiveness of occupational therapy interventions on function and satisfaction with occupational performance among adults with conditions of the hand, wrist, and forearm: a protocol for a systematic review

**DOI:** 10.12688/hrbopenres.13584.1

**Published:** 2022-08-15

**Authors:** Margo Sheerin, Cliona O' Riordan, Mairead Conneely, Leonora Carey, Damien Ryan, Rose Galvin, Ann-Marie Morrissey

**Affiliations:** 1 Department of Occupational Therapy, University of Limerick Hospital Group, Dooradoyle, Limerick, Ireland; 2 School of Allied Health, Faculty of Education and Health Sciences, Ageing Research Centre, Health Research Institute, University of Limerick, Limerick, Ireland; 3 ALERT- Limerick EM Education Research Training, Emergency Department,, University Hospital Limerick, Dooradoyle, Limerick, Ireland

**Keywords:** Occupational therapy, hand, wrist, forearm

## Abstract

**Background:** Functional hand use post injury is important in enabling a person’s engagement in daily living tasks. Without proper treatment, there may be difficulties in self-care, engaging in job roles, or leisure pursuits. Occupational therapists are key health care practitioners for people with upper limb conditions. This systematic review aims to appraise and summarise current evidence regarding effectiveness of occupational therapy interventions among adults with conditions of the hand, wrist, and forearm.

**Methods:** A systematic review of randomised control trials and quasi randomised controlled trials will be completed. MEDLINE OVID, CINAHL, CENTRAL, COCHRANE, PUBMED and EMBASE databases will be systematically searched. Grey literature will be searched for via Google Scholar. Studies will be included if they include provision of occupational therapy to adults with a hand, wrist, or forearm condition when compared to treatment as usual or an alternative treatment option. The primary outcome will be function. Secondary outcomes will include satisfaction with occupational performance, quality of life, pain experience, and participation. The Brief International Classification of Functioning, Disability and Health (ICF) Core Set for Hand Conditions will be used to categorise outcomes. The Cochrane Risk of Bias 2 (RoB 2) tool and the Grading of Recommendations Assessment, Development, and Evaluations (GRADE) framework will be utilised to assess quality. A pooled meta- analysis will be completed using RevMan, depending on the uniformity and availability of data.

**Results**: This review aims to synthesise high quality evidence to identify the effectiveness of occupational therapy interventions with patients with a hand, wrist, or forearm condition, categorising outcomes in relation to the ICF Core Set for Hand Conditions.

**Conclusions**: By synthesising the evidence there is potential for improved evidence base for clinicians; improved outcomes for patients; as well as potential economic benefit.

This study is registered with PROSPERO: CRD42022337070.

## Introduction

Hand and wrist injuries are a common occurrence, estimated to account for between 10% and 30% of all emergency department presentations ^1– 3^. Most uncomplicated injuries will recover fully, however, accurate assessment and treatment are vital as mismanagement can result in a delayed recovery and is associated with large individual and societal costs ^3– 5^. It is acknowledged within the literature that individuals with a hand and wrist condition experience reduced functional performance including difficulty with self-care, mobility, housekeeping activities, work, as well as economic and social implications ^6– 9^ and correlations with anxiety, depression, and reduced quality of life ^10^. 

Occupational therapists are key health care practitioners in assessing and treating people with an upper limb condition, as occupational therapy’s primary goal is to facilitate adjustments to lifestyle and to prevent loss of function ^11^. The Hand Therapy Certification Commission ^12^ reports that 87% of their members who are certified hand therapists (CHT) are occupational therapists, with the remaining 12% physical therapists, and 1% having both occupational therapy and physical therapy qualifications. The establishment of hand therapy clinics, commonly led by occupational therapists, is an area which is gaining momentum within the literature as being cost effective, enabling specialised early intervention, and reduced surgeon workload ^13– 15^.

Occupational therapists offer a range of interventions in treating the upper limb including splinting, targeted exercises, occupation-based interventions, oedema management, activity modification, and scar management. The importance of looking beyond biomechanics, to an individual’s ability to function, including *activity*, or engagement in a task or action, and *participation*, or involvement in a situation, is brought to the fore in the International Classification of Functioning, Disability and Health (ICF) ^16^ which describes a person’s health and their lived experience of health, with a focus on the persons functioning and engagement with their environment ^17^. In 2012, the ICF Core Set for Hand Conditions were developed to best describe function and disability for those with a hand condition, including activity and participation categories such as carrying out a daily routine ^18, 19^. This framework links closely with occupational therapy theory and challenges therapists working with hand conditions to consider the person as an occupational being. A systematic review of 59 observational and experimental studies by Roll and Hardison ^20^, explored the evidence in relation to occupational therapy interventions for musculoskeletal disorders of the upper limb published between 2006 and 2014. Heterogeneity across the study protocols limited the ability of the authors to identify the clinical impact of any one specific intervention approach. However, the strongest evidence across the included studies supported postsurgical early active motion protocols and splinting for various conditions ^20^. Few studies explored occupation-based interventions or outcomes relating to patient satisfaction and quality of life. A number of experimental studies have been published since this review that explore the impact of occupational therapy interventions on upper limb function. The current study aims to locate, appraise and synthesise current evidence regarding effectiveness of occupational therapy for adults presenting with a musculoskeletal condition of the hand, wrist, or forearm. The Brief ICF Core Set for Hand Conditions will be used to categorise outcomes, with the purpose of capturing the impact of occupational therapy interventions on a person’s ability to function and participate within their environment. 

## Methods

### Study design

This protocol for a systematic review is conducted and reported in accordance with the Preferred Reporting Items for Systematic Review and Meta-Analysis Protocols (PRISMA-P) 2015 statement ^21, 22^. The systematic review and meta-analyses will comply with the reporting guidance outlined in PRISMA checklist ^22^. The methodology for the review will be guided by the Cochrane Handbook for Systematic Reviews of Interventions ^23^. 

### Search strategy

Searches will be carried out in CINAHL (1984), Cochrane Central Register of Controlled Trials (CENTRAL) (1996), Cochrane Database of Systematic Reviews (CDSR) (1995), MEDLINE (OVID) (1946), EMBASE (1974), and PubMed (1996). MeSH terms and associated key words will be used covering the topics of hand, wrist, and forearm injury or condition, adults, occupational therapy, hand therapy, occupational performance, satisfaction, and function. Grey literature will be searched for via Google Scholar. The reference lists of all eligible studies will be hand searched for further relevant studies.

### Eligibility criteria

***Population*.** Adults (>18 years) with a hand, wrist, or forearm condition or injury which is limiting function.

***Intervention*.** Intervention provided by an occupational therapist, or within the occupational therapist’s scope of practice, to treat hand, wrist, or forearm conditions resulting from trauma, disease, congenital or acquired deformity. Interventions include: occupation based intervention, activity programmes, range of movement (ROM) programmes, customised orthotic or splint fabrication, management of pain, strengthening programmes, oedema management, wound management, and scar care ^24^.

***Comparison*.** Usual care as defined by the study authors or another active intervention.

***Outcome*.** The primary outcome will be patient functional ability post occupational therapy intervention, measured using a validated scale, such as the Disabilities Assessment of Shoulder and Hand (DASH) ^25^ and Patient-Rated Wrist Hand Evaluation (PRWHE) ^26^. Secondary outcomes include patient satisfaction, pain experience, activity limitation and quality of life using outcome measures such as the Canadian Occupational Performance Measure (COPM) ^27^ and WHOQOL BREF ^28^. The ICF Core Set for Hand Conditions will be used to categorise outcomes. Within this classification, the components of Body Functions (b), Body Structures (s), Activities and Participation (d) and Environmental Factors (e) will be used to categorise occupational therapy interventions reported on within this review. To ensure scientific rigour, only trials with a randomised design will be included. Randomised Control Trials (RCTs) and quasi-RCTs will be included in this review.

All available papers will be searched, with no restriction on year of publication. Non-English language studies will be included in the review and translation to English will be sought for articles retrieved from database searches via Google translate.

### Exclusion criteria

Studies will be excluded if their population is <18 years; if participants present with specific complex medical complaints, for example cerebral vascular accident (CVA), where the primary presentation is not in relation to the hand condition; studies targeting the shoulder or elbow and not the distal upper limb; and where the primary treating clinician providing intervention is not an occupational therapist or interventions are not within an occupational therapist’s scope of practice ^24^. 

### Study selection and data extraction

***Screening*.** References generated from the search will be exported to Endnote X9 software, where duplicates will be deleted. Alternative unpaid software such as Zotero or Qigga could be used to manage references and share research. Two authors (MS and COR) will independently screen the studies by title and abstract for eligibility. Studies that are selected by the reviewers as meeting inclusion criteria will undergo a full text review. Should a disagreement regarding eligibility arise, both authors will meet to come to a consensus. Where consensus cannot be reached between the two authors, third and fourth authors (RG and DR) will be consulted.

***Study synthesis and analysis*.** Data will be extracted from the relevant studies by one reviewer (MS). A second author (RG) will independently check 20% of data extracted. The information extracted will include authors, year of publication, country, setting, study sample size, study design, outcome measures, and period of follow-up. Data will be collated into a prepared Microsoft Excel 2016 Version 16.0 document. A meta-analysis will be completed where the data are homogenous, which will be determined by the outcomes measured and the time points accessed across the included studies. For the primary outcome of function, risk ratios will be calculated with a 95% CI to determine the intervention effect. The same approach will apply to all secondary outcome measures. For continuous data, mean differences with 95% CIs will be reported or standardised mean difference (SMD) and 95% CI will be applied where studies used different methods of measurement. The median and IQR will be used in studies where mean and SD are not reported ^29^. Where study data are not available, the authors will be contacted. Heterogeneity will be explored by visually inspecting the forest plots and the associated I ^2^ statistics. We will consider an I ^2^ >50% as significant heterogeneity. Where there is evidence of significant heterogeneity, we will conduct a meta-analysis using both a fixed-effect (FEM) and random-effects model (REM) and we will present the most conservative outcome ^30^. RevMan V.5.4.1 software will be used to analyse the data for the meta-analysis.

***Quality assessment and certainty of evidence*.** Studies that meet the inclusion criteria will be assessed for risk of bias using the Cochrane Risk of Bias 2 (RoB 2) tool ^31^. Two independent reviewers (MS and MC) will assess the studies’ risk of bias for selection bias, performance bias, attrition bias, reporting bias, outcomes, and overall risk of bias. The Grading of Recommendations, Assessment, and Development and Evaluations framework (GRADE) will be used to determine the overall certainty of evidence for outcomes reported ^32^. Outcomes will be graded at one of four levels of evidence, namely, very low certainty, low certainty, moderate certainty, and high certainty ^33^.

## Ethics and dissemination

Formal ethical approval is not required for the literature review as all data collected will be secondary data. The findings of this review will be disseminated through publication in a peer-reviewed journal, presentation at relevant conferences and meetings of professional associations, presenting programme results to local community groups and other local stakeholders, sharing information through social media or on the hospital group website, and summarising findings in progress reports for funders.

## Discussion

This review will update and synthesise the totality of evidence relating to the effectiveness of occupational therapy interventions on functional outcomes for adults with a hand, wrist, or forearm musculoskeletal condition. It aims to further explore and report on outcomes in relation to activity limitations, participation restrictions, satisfaction with occupational performance, pain experience, and quality of life.

A variety of techniques and tools may be used in therapeutic intervention with the hand and upper limb. It is anticipated that this review will identify the components of occupational therapy interventions utilised with patients with a hand, wrist, or forearm condition and their benefits. This will have relevance for clinicians and policy makers by enabling recommendations for occupational therapy for those post hand, wrist, and forearm injury in line with evidenced based practice.

This review will categorise outcomes in relation to the Brief ICF Core Set for Hand Conditions to best describe functional consequences for those with a musculoskeletal distal upper limb condition, including activity and participation. This may further inform therapists working with hand conditions on the value of providing client- centred meaningful interventions to promote recovery, participation, and quality of life.

Strengths of this review will include the use of stringent methods, such as PRISMA-P guidelines and the GRADE framework. Limitations may include a high level of heterogeneity in the included studies. This may affect the ability to complete a meta-analysis.

## Study status

Database searches have been completed.

## Data availability

### Underlying data

No underlying data are associated with this article.

### Extended data

figshare: Supporting Documentation: Effectiveness of occupational therapy interventions on function and satisfaction with occupational performance among adults with conditions of the hand, wrist, and forearm: a protocol for a systematic review. https://doi.org/10.6084/m9.figshare.20398989.v1 ^33^

### Reporting guidelines

figshare: PRISMA-P checklist for ‘Effectiveness of occupational therapy interventions on function and satisfaction with occupational performance among adults with conditions of the hand, wrist, and forearm: a protocol for a systematic review’. https://doi.org/10.6084/m9.figshare.20398989.v1 ^33^
