# Supplementary material for: Effectiveness of occupational therapy interventions on function and satisfaction with occupational performance among adults with conditions of the hand, wrist, and forearm: a protocol for a systematic review
Source: HRB Open Res. 2024 Feb 12;5:56. Originally published 2022 Aug 15. [Version 2] doi: 10.12688/hrbopenres.13584.2 (PMC10897503; doi:10.12688/hrbopenres.13584.2)
Supplement: Peer review history

# Reviewer report: Reviewer response for version 2

**DOI:** 10.21956/hrbopenres.15201.r38188

**Published:** 2024-02-26

**Authors:** Deirdre Connolly

**Affiliations:** 1 Trinity St. James’s Cancer Institute, Dublin, Ireland

I have reviewed this protocol and am satisfied that all revisions have been made. I recommend this protocol for publication.

Is the study design appropriate for the research question?

Yes

Is the rationale for, and objectives of, the study clearly described?

Yes

Are sufficient details of the methods provided to allow replication by others?

Partly

Are the datasets clearly presented in a useable and accessible format?

Not applicable

Reviewer Expertise:

Self-management interventions for Inflammatory Arthritis

I confirm that I have read this submission and believe that I have an appropriate level of expertise to confirm that it is of an acceptable scientific standard.

# Reviewer report: Reviewer response for version 1

**DOI:** 10.21956/hrbopenres.14838.r35712

**Published:** 2023-10-13

**Authors:** Deirdre Connolly

**Affiliations:** 1 Trinity St. James’s Cancer Institute, Dublin, Ireland

This review will examine effectiveness of occupational therapy (OT) interventions for people with conditions of hand, wrist and forearm. This is a common area of OT practice and will add to the evidence-base in this area. 

 The methods for the review are well described. Some suggestions for consideration include:

 Why have the authors not included conditions of the elbow and shoulder? There is no explicit rationale for excluding these joints.

 More detail is needed on the level/sources of grey literature to be included in the search strategy.

 Intervention: Authors state inclusion of "occupation based interventions" but have not clearly defined what these include in relation to conditions of hand, wrist and forearm. Interventions do not include fatigue which is a regular area of OT intervention for people with Inflammatory Arthritis.

Is the study design appropriate for the research question?

Yes

Is the rationale for, and objectives of, the study clearly described?

Yes

Are sufficient details of the methods provided to allow replication by others?

Partly

Are the datasets clearly presented in a useable and accessible format?

Not applicable

Reviewer Expertise:

Self-management interventions for Inflammatory Arthritis

I confirm that I have read this submission and believe that I have an appropriate level of expertise to confirm that it is of an acceptable scientific standard, however I have significant reservations, as outlined above.

# Reviewer report: Reviewer response for version 1

**DOI:** 10.21956/hrbopenres.14838.r32680

**Published:** 2022-09-07

**Authors:** Tadhg Stapleton

**Affiliations:** 1 Discipline of Occupational Therapy, School of Medicine, Trinity College Dublin, Dublin, Ireland

- Consider how different UL injuries and different OM usage in the selected papers may impact on the planned meta-analysis?
- Unsure if grey literature will yield any useful articles as the plan is to only include trials with randomised design?

These can be addressed by the authors during the completion of the proposed systematic review.

Is the study design appropriate for the research question?

Yes

Is the rationale for, and objectives of, the study clearly described?

Yes

Are sufficient details of the methods provided to allow replication by others?

Yes

Are the datasets clearly presented in a useable and accessible format?

Not applicable

Reviewer Expertise:

Applied clinical-based research in areas of occupational therapy interventions including hand injuries

I confirm that I have read this submission and believe that I have an appropriate level of expertise to confirm that it is of an acceptable scientific standard.
